# Overexpression of patatin-related phospholipase AIIIβ altered the content and composition of sphingolipids in *Arabidopsis*

**DOI:** 10.3389/fpls.2014.00553

**Published:** 2014-10-21

**Authors:** Maoyin Li, Jonathan E. Markham, Xuemin Wang

**Affiliations:** ^1^Department of Biology, University of MissouriSt. Louis, MO, USA; ^2^Donald Danforth Plant Science CenterSt. Louis, MO, USA; ^3^Department of Biochemistry, University of Nebraska-LincolnLincoln, NE, USA

**Keywords:** *Arabidopsis thaliana*, patatin-related phospholipase, sphingolipid, plant growth, fatty acyl flux

## Abstract

In plants, fatty acids are primarily synthesized in plastids and then transported to the endoplasmic reticulum (ER) for synthesis of most of the complex membrane lipids, including glycerolipids and sphingolipids. The first step of sphingolipid synthesis, which uses a fatty acid and a serine as substrates, is critical for sphingolipid homeostasis; its disruption leads to an altered plant growth. Phospholipase As have been implicated in the trafficking of fatty acids from plastids to the ER. Previously, we found that overexpression of a patatin-related phospholipase, *pPLAIII*β, resulted in a smaller plant size and altered anisotropic cell expansion. Here, we determined the content and composition of sphingolipids in *pPLAIII*β-knockout and overexpression plants (*pPLAIII*β*-KO* and *-OE*). 3-keto-sphinganine, the product of the first step of sphingolipid synthesis, had a 26% decrease in leaves of *pPLAIII*β*-KO* while a 52% increase in *pPLAIII*β*-OE* compared to wild type (WT). The levels of free long-chain base species, dihydroxy-C18:0 and trihydroxy-18:0 (d18:0 and t18:0), were 38 and 97% higher, respectively, in *pPLAIII*β*-OE* than in WT. The level of complex sphingolipids ceramide d18:0–16:0 and t18:1–16:0 had a twofold increase in *pPLAIII*β*-OE*. The level of hydroxy ceramide d18:0–h16:0 was 72% higher in *pPLAIII*β*-OE* compared to WT. The levels of several species of glucosylceramide and glycosylinositolphosphoceramide tended to be higher in *pPLAIII*β*-OE* than in WT. The total content of the complex sphingolipids showed a slightly higher in *pPLAIII*β*-OE* than in WT. These results revealed an involvement of phospholipase-mediated lipid homeostasis in plant growth.

## INTRODUCTION

Lipids are structural components of membrane bilayers and play important metabolic and regulatory roles in plant growth, development, and stress responses. Phospholipases are major enzyme families that catalyze many of the reactions in lipid metabolism and signaling. Recently, multiple biological functions have been revealed for patatin-related phospholipase As (pPLAs; Li and Wang, 2014). Patatin-related PLAs in *Arabidopsis* comprise pPLAI, pPLAII (α,β,γ,δ,𝜀), and pPLAIII (α,β,γ,δ; Scherer et al., 2010). *pPLAI* has a positive role in plant resistance to the fungus pathogen *Botrytis cinerea*, possibly by mediating the production of jasmonates (Yang et al., 2007). Deficiency of *pPLAII*α decreases resistance to bacterial pathogens and impedes oxylipin production under drought stress (La Camera et al., 2005; Yang et al., 2012). *pPLAII*γ, *pPLAII*δ, and *pPLAII*𝜀 are involved in the response to phosphorus deficiency and auxin treatment in terms of root elongation (Rietz et al., 2004, 2010).

pPLAIIIs possess a distinctive non-canonical esterase motif GxGxG, instead of GxSxG, which is present in pPLAI and pPLAIIs (Scherer et al., 2010). Overexpression of *pPLAIII*δ leads to a stunted plant statue (Huang et al., 2001). Overexpression of *pPLAIII*β results in smaller plant size and reduced cellulose content in stems (Li et al., 2011). Disruption of rice *DEP3*, a homolog of *pPLAIII*δ, results in taller rice plants (Qiao et al., 2011). Heterogeneous overexpression of an *Oncidium OSAG78*, another homolog of *pPLAIII*δ, results in a smaller plant size and a delayed flowering time in *Arabidopsis* (Lin et al., 2011). These lines of evidence indicate *pPLAIIIs* are important for plant growth and development.

In plants, sphingolipids are major components of cellular membranes and determine the membrane physical properties. They have functions on environmental stress tolerance (Chao et al., 2011; Chen et al., 2012), programmed cell death (Alden et al., 2011), and polar auxin transport (Markham et al., 2011; Yang et al., 2013). Sphingolipids include free long chain bases, such as long chain bases (LCBs) and long chain base phosphate (LCBPs), and complex sphingolipids, such as ceramide (Cer), hydroxyceramide (hCer), glucosylceramide (GlcCer), and glycosylinositolphosphoceramide (GIPC; Markham et al., 2013). Sphingolipid synthesis begins by the condensation of palmitoyl-CoA and serine catalyzed by serine palmitoyltransferase (SPT; Hanada, 2003). SPT are heterodimer proteins with two subunits, LCB1 and LCB2. In *Arabidopsis*, LCB1 is encoded by a single gene (Chen et al., 2006), while LCB2 is encoded by two functionally redundant genes, *LCB2a* and *LCB2b* (Dietrich et al., 2008).

Maintenance of sphingolipid homeostasis is critical for plant growth and development (Chen et al., 2006; Dietrich et al., 2008; Teng et al., 2008; Kimberlin et al., 2013). T-DNA disruption of *LCB1* gene in *Arabidopsis* results in an arrested development of the embryo at the globular stage (Chen et al., 2006). Partial RNA interference suppression of *LCB1* results in reduced cell expansion, a smaller plant, and elevated levels of saturated sphingolipid LCBs (Chen et al., 2006). There is no apparent growth phenotype for mutants deficient in either *LCB2a* or *LCB2b*, however, the deficiency of both is lethal for gametophyte (Dietrich et al., 2008). Inducible suppression of *LCB2b* results in cell necrosis and reduced levels of LCBs in adult *Arabidopsis* plants (Dietrich et al., 2008).

The function of SPT can be regulated by small polypeptides designated as small subunits of SPT (ssSPT). ssSPTa and ssSPTb interact with SPT and stimulate its activity in *Arabidopsis* (Kimberlin et al., 2013). T-DNA disruption of *ssSPTa* results in reduced plant growth and pollen lethality in *Arabidopsis* (Kimberlin et al., 2013). Overexpression of *ssSPTa* leads to increased levels of free LCBs and LCBPs compared with that of WT, while RNA interference suppression of *ssSPTa* has opposite effects (Kimberlin et al., 2013). Overexpression of *ssSPTa* results in a greater reduction in plant growth than suppression does, when plants are treated by fumonisin B1, an inhibitor of sphingolipid synthesis (Kimberlin et al., 2013).

Previously, we reported that overexpression of *pPLAIII*β results in a reduced plant growth in *Arabidopsis* (Li et al., 2011). Here we report the effects of overexpression of *pPLAIII*β on the content and composition of sphingolipids, including free sphingolipids and complex ones. Our results show that overexpression of *pPLAIII*β results in an elevated level of 3-keto-sphingaine (3-KS), the product of the first step of sphingolipid synthesis, as well as altered levels of many of the species of complex sphingolipids.

## RESULTS

### OVEREXPRESSION OF *pPLAIII*β INCREASED LEVELS OF 3-KETO-SPHINGANINE AND FREE LONG-CHAIN BASES

**FIGURE 1:**
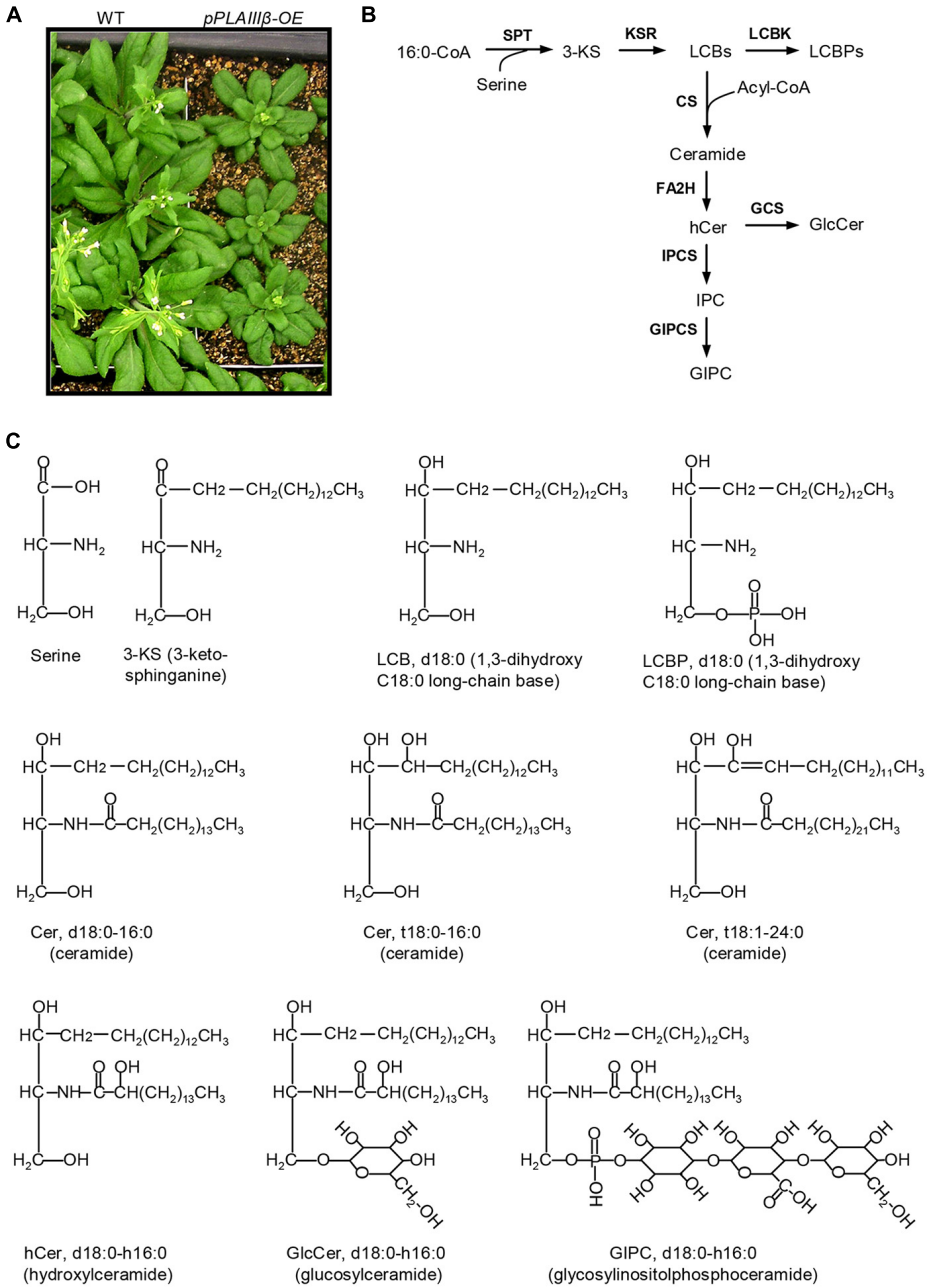
**Schematic representation of sphingolipid biosynthesis in *Arabidopsis*. (A)** *pPLAIII*β-overexpressing mutants (β-OE) were smaller than wild type (WT). **(B)** Representative diagram of the sphingolipid biosynthesis pathways (Markham et al., 2013). **(C)** Representative sphingolipid molecules. 3-KS, 3-keto-sphinganine; Cer, ceramide; CS, ceramide synthase; FA2H, fatty acid 2-hydroxylase; GCS, glucosylceramide synthase; GIPC, glycosylinositolphosphoceramide; GIPCS, glycosylinositolphosphoceramide synthase; GlcCer, glucosylceramide; hCer, hydroxyceramide; IPC, inositolphosphoceramide; IPCS, inositolphosphoceramide synthase; KSR, 3-ketosphinganine reductase; LCBK, long-chain base kinase; LCBP, LCB phosphate; LCBs, long-chain bases; SPT, serine-palmitoyltransferase.

Overexpression of *pPLAIII*β by constitutive 35S cauliflower mosaic virus promoter in *Arabidopsis* resulted in stunted plant growth (**Figure 1A**). The sphingolipids were profiled in leaves of WT, *pPLAIII*β-knockout (β*-KO*), and *pPLAIII*β-overexpressors (β*-OE*). The first step of sphingolipid synthesis is the production of 3-KS catalyzed by the serine palmitoyltransferase using substrates of 16:0-CoA and serine (**Figure 1B**). The reduction of 3-KS forms a dihydroxy C18 long chain base (sphinganine), designated as LCB d18:0 (**Figures 1B,C**). The reduction of LCB d18:0 forms the trihydroxy LCB (phytosphingosine), designated as LCB t18:0. Desaturation of LCB d18:0 and t18:0 produces LCB d18:1 and t18:1. The LCB d18:0, d18:1, t18:0, and t18:1 can be phosphorylated to form LCBP d18:0, d18:1, t18:0, and t18:1(**Figures 1B,C**). LCBs and LCBPs belong to free sphingolipids.

**FIGURE 2:**
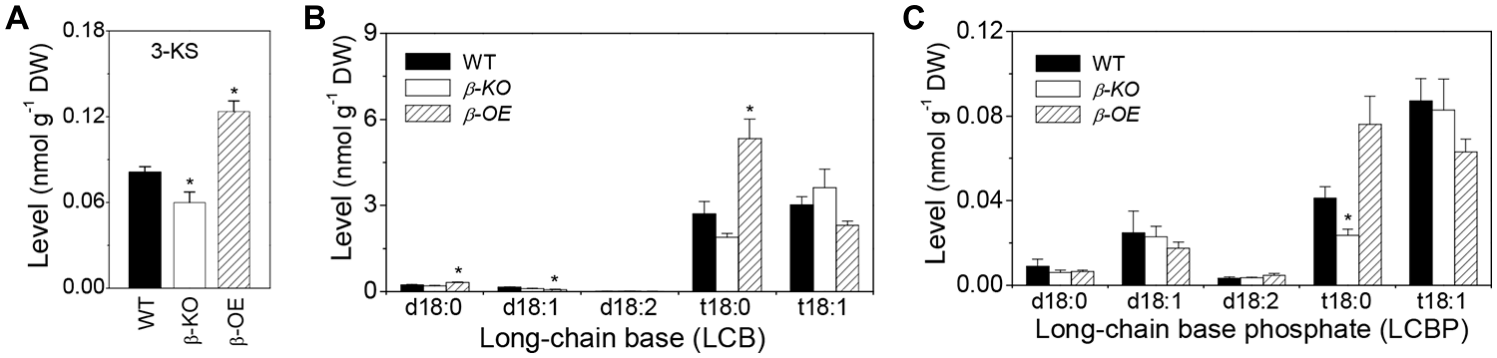
**Levels of 3-KS, LCBs, and LCBP in *pPLAIII*β-knockout and overexpression plant leaves. (A)** The level of 3-keto-sphingaine (3-KS). **(B)** The level of long-chain bases (LCBs). **(C)** The level of long chain base-phosphates (LCBPs). β*-KO*, T-DNA knockout of *pPLAIII*β (Salk_057212). β*-OE*, overexpression mutant of *pPLAIII*β driven by cauliflower mosaic 35S promoter. Values are means ± SE (*n* = 5). *Significant difference at *P* < 0.05 compared with the WT, based on Student’s *t*-test.

The level of 3-KS was 26% lower in β*-KO* and 52% higher in β*-OE* compared with that of WT (**Figure 2A**). The levels of LCB t18:0 and t18:1 were approximately 15 times higher than LCB d18:0 and d18:1 in leaves of WT (**Figure 2B**). Among the LCB species, the levels of d18:0 and t18:0 were 38 and 97% higher, respectively, in leaves of β*-OE* compared to those of WT (**Figure 2B**). The level of LCB t18:0 tended to be lower in β*-KO* than in WT (**Figure 2B**). Of the LCBP species, the level of t18:0 tended to be 85% higher while it was 43% lower in β*-KO* than in WT (**Figure 2C**).

### OVEREXPRESSION OF *pPLAIII*β ALTERED THE LEVELS OF CERAMIDE AND HYDROXYCERAMIDE

**FIGURE 3:**
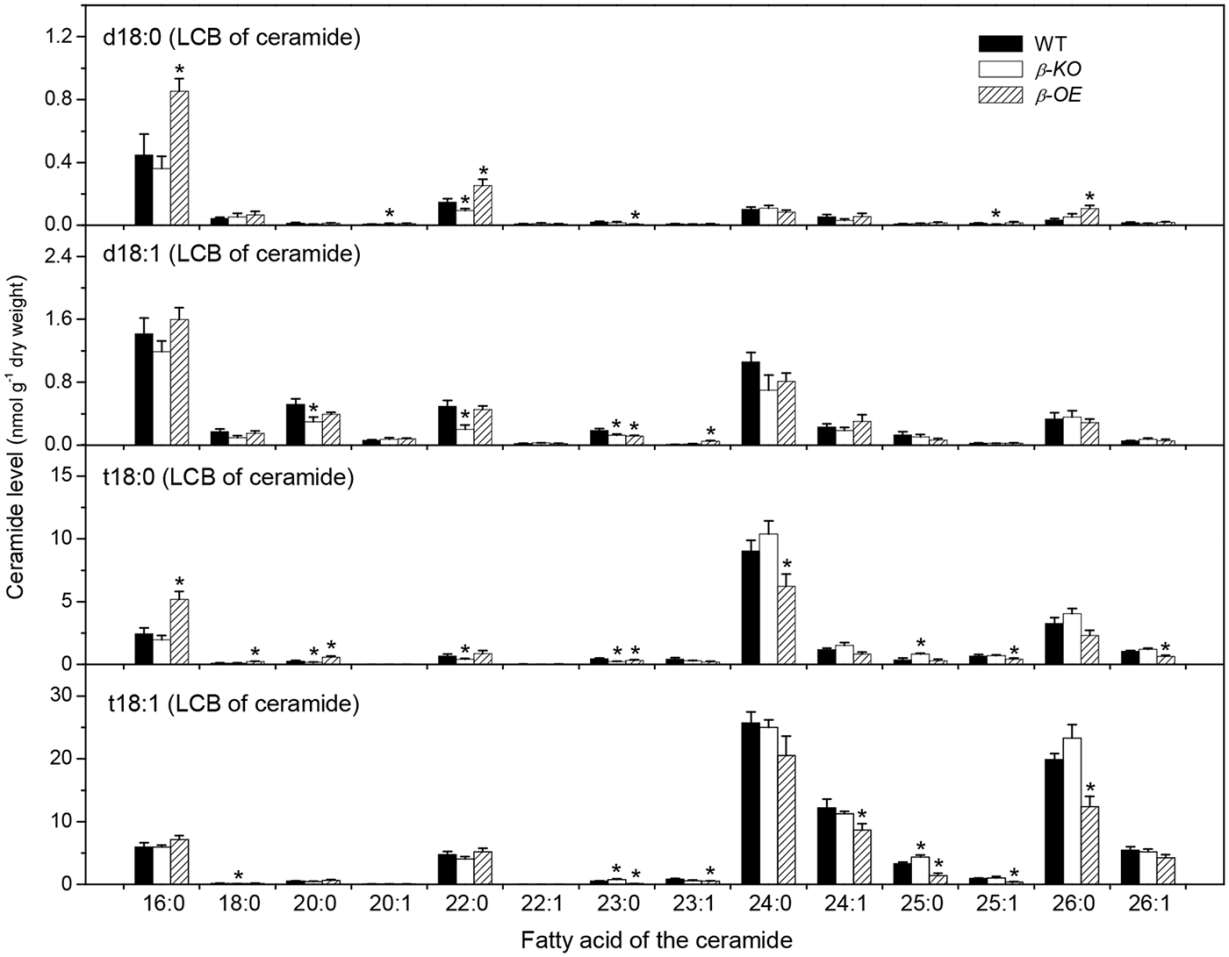
**Levels of Cer species in *pPLAIII*β-knockout and overexpression plant leaves.** β*-KO*, T-DNA knockout of *pPLAIII*β (Salk_057212). β*-OE*, overexpression mutant of *pPLAIII*β driven by cauliflower mosaic 35S promoter. Values are means ± SE (*n* = 5). *Significant difference at *P* < 0.05 compared with the WT, based on Student’s *t*-test.

Ceramide was synthesized by CS using substrates of LCBs and acyl-CoAs (**Figure 1B**). A Cer molecule contains two components, a LCB and a fatty acid chain, linked by an amide bond. For example, Cer d18:0–16:0 comprises a LCB d18:0 and a fatty acyl chain 16:0 (**Figure 1C**). The levels of Cer molecules containing one of four types of LCBs and one of the 14 types of fatty acyl chains were quantified by mass spectrometry (**Figure 3**). The four types of LCBs are d18:0, d18:0, t18:0, and t18:1, and the most abundant fatty acyl chains are 16:0, 22:0, 24:0, and 26:0 (**Figure 3**). The levels of 16:0-containing Cers, including d18:0–16:0, d18:1–16:0, t18:0–16:0, and t18:1–16:0, tended to be lower in β*-KO* while higher in β*-OE* than in WT (**Figure 3**). The levels of Cer d18:0–16:0 and t18:0–16:0 were about twofold higher in β*-OE* than in WT (**Figure 3**). The levels of 24:0-, 24:1-, and 26:0-containing Cers, including t18:0–24:0, t18:1–24:1, and t18:1–26:0, tended to be lower in β*-OE* than in WT (**Figure 3**). Generally the levels of Cers containing fatty acyl chain of 16–22 carbons tended to be higher while those containing fatty acyl chain of 24–26 carbons tended to be lower in leaves of β*-OE* than in WT, and the β*-KO* behaved oppositely (**Figure 3**).

**FIGURE 4:**
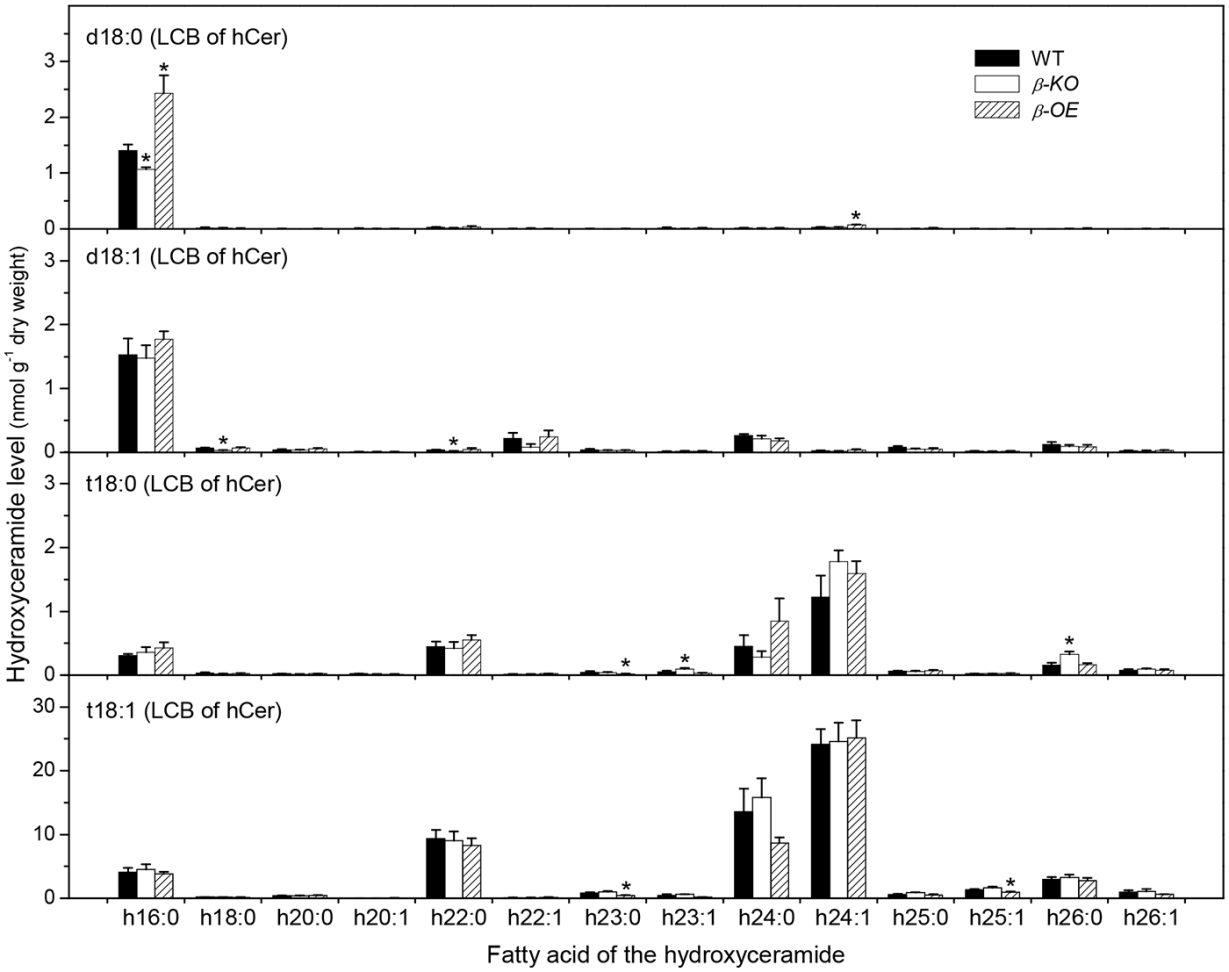
**Levels of hCer species in *pPLAIII*β-knockout and overexpression plant leaves.** β*-KO*, T-DNA knockout of *pPLAIII*β (Salk_057212). β*-OE*, overexpression mutant of *pPLAIII*β driven by cauliflower mosaic 35S promoter. Values are means ± SE (*n* = 5). *Significant difference at *P* < 0.05 compared with the WT, based on Student’s *t*-test.

The fatty acyl chains of Cer can be hydroxylated to produce hydroxyl ceramide (hCer; **Figure 1B**). For example, the hydroxylation of 16:0 in Cer d18:0–16:0 led to the formation of hCer d18:0–h16:0 (**Figure 1C**). The levels of hCer species containing one of the four types of LCBs and one the 14 types of hydroxylated fatty acyl chains were profiled (**Figure 4**). The most profound alteration was the level of hCer d18:0–h16:0; it was 24% lower in β*-KO* and 72% higher in β*-OE* than in WT (**Figure 4**). The levels of the other hCer species did not display any significant alteration between WT and β*-OE* (**Figure 4**).

### OVEREXPRESSION OF *pPLAIII*β CHANGED THE LEVELS OF GLUCOSYLCERAMIDE AND GLYCOSYLINOSITOLPHOSPHOCERAMIDE

**FIGURE 5:**
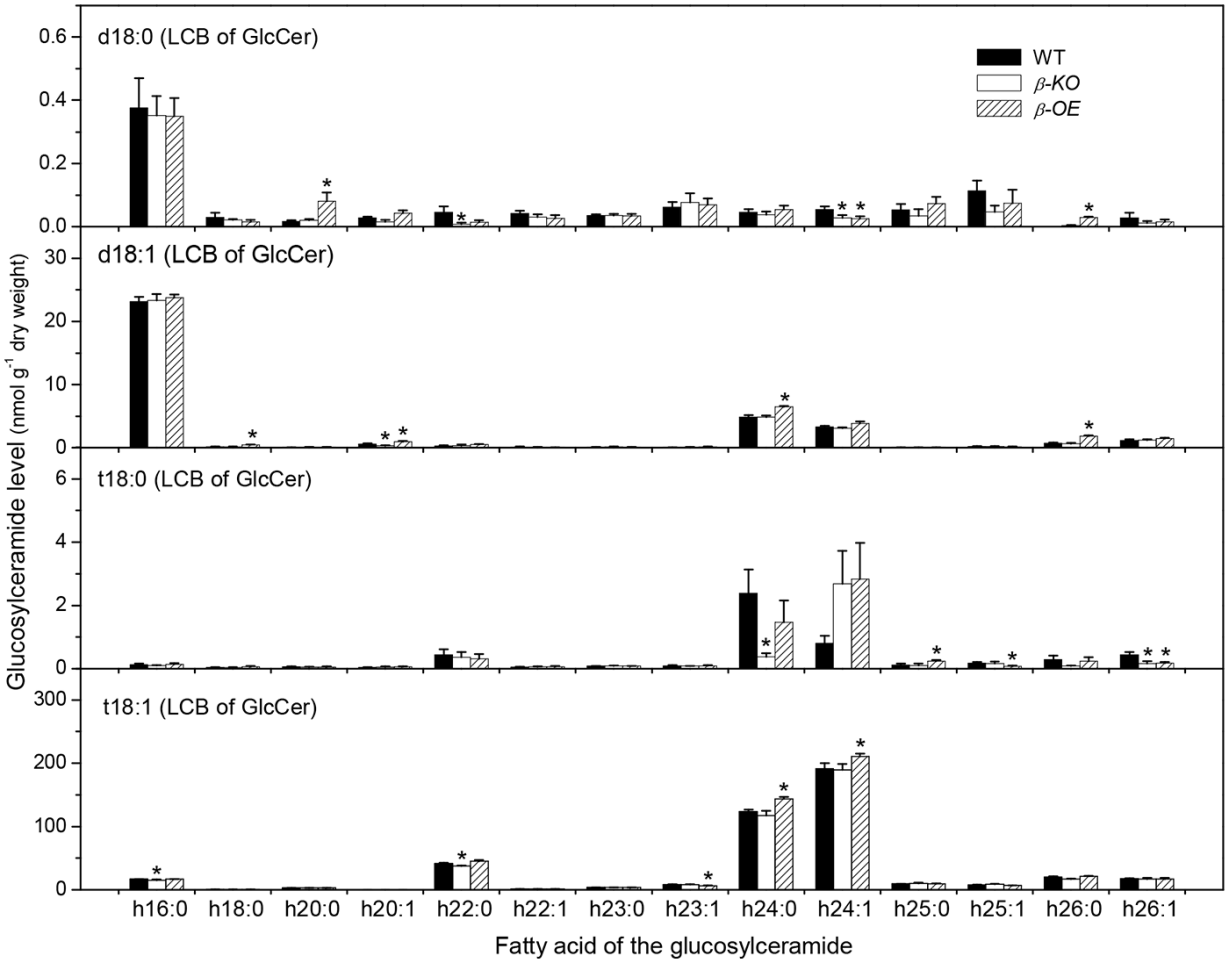
**Levels of GlcCer species in *pPLAIII*β-knockout and overexpression plant leaves.** β*-KO*, T-DNA knockout of *pPLAIII*β (Salk_057212). β*-OE*, overexpression mutant of *pPLAIII*β driven by cauliflower mosaic 35S promoter. Values are means ± SE (*n* = 5). *Significant difference at *P* < 0.05 compared with the WT, based on Student’s *t*-test.

**FIGURE 6:**
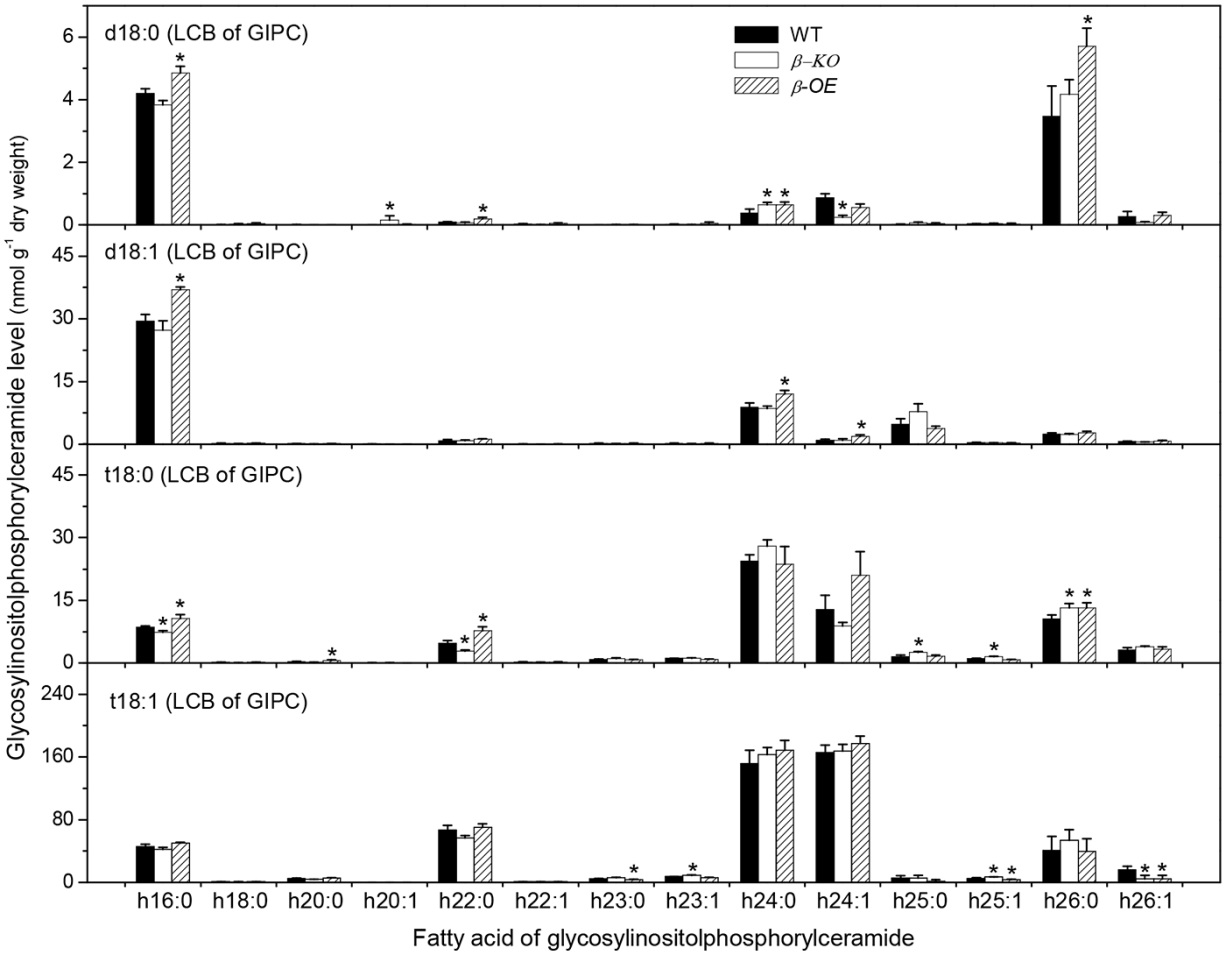
**Levels of GIPC species in *pPLAIII*β-knockout and overexpression plant leaves.** β*-KO*, T-DNA knockout of *pPLAIII*β (Salk_057212). β*-OE*, overexpression mutant of *pPLAIII*β driven by cauliflower mosaic 35S promoter. Values are means ± SE (*n* = 5). *Significant difference at *P* < 0.05 compared with the WT, based on Student’s *t*-test.

A sugar-containing polar head group can be linked to the hydroxyl ceramide to form GlcCer and GIPC (**Figure 1B**). For example, GluCer d18:0–h16:0 has a glycosyl head group and GIPC d18:0–h16:0 has a phosphoryl-inositol-hexose-hexuronic acid head group (**Figure 1C**). Some GlcCer species displayed higher levels in leaves of β*-OE* than in WT, including GlcCer d18:0–h20:0, d18:1–h24:0, t18:1–h24:0, and t18:1–h24:1 (**Figure 5**). The level of GlcCer t18:0–h24:0 was 80% lower in β*-KO* (**Figure 5**). The profound alteration of GIPC species was d18:0–h26:0; its levels increased 64% in β*-OE* compared to WT (**Figure 6**). The levels of GIPC d18:0–h16:0, d18:1–h16:0, and t18:0–h16:0 tended to be lower in β*-KO* while higher in β*-OE* than in WT (**Figure 6**). Generally most of the GIPC species tended to be lower in β*-KO* and higher in β*-OE* than in WT (**Figure 6**).

**FIGURE 7:**
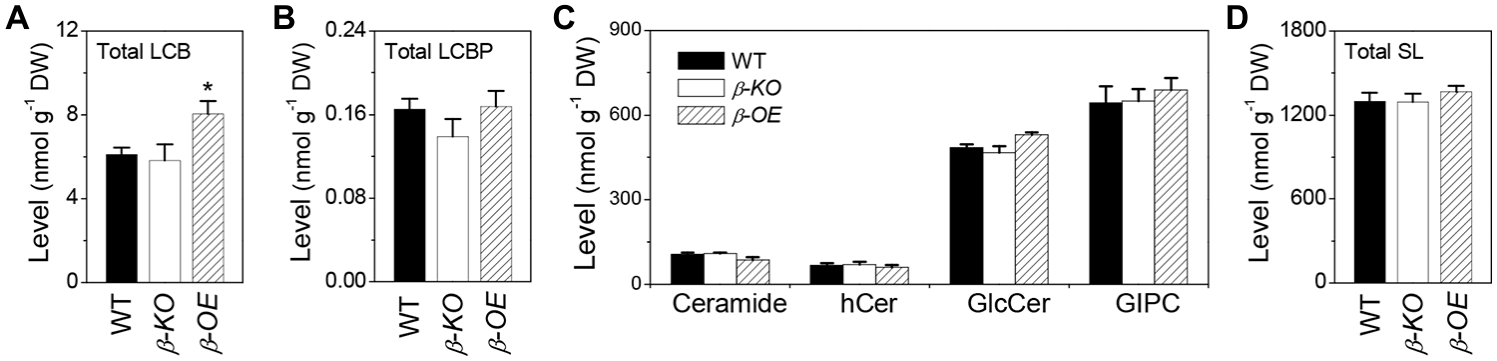
**Levels of total LCBs, total LCBPs, and total complex sphingolipids in *pPLAIII*β-knockout and overexpression plant leaves. (A)** The level of total LCBs, summarized from individual species in **Figure 2B**. **(B)** The level of total LCBPs, summarized from individual species in **Figure 2C**. **(C)** The levels of total complex sphingolipids, including Cer, hCer, GlcCer, and GIPC, summarized from individual species in **Figures 3–6**. **(D)** The total content of sphingolipids (SL), including free sphingolipids, such as LCBs and LCBPs, and complex sphingolipids, such as Cer, hCer, GlcCer, and GIPC. β*-KO*, T-DNA knockout of *pPLAIII*β (Salk_057212). β*-OE*, overexpression mutant of *pPLAIII*β driven by cauliflower mosaic 35S promoter. Values are means ± SE (*n* = 5). *Significant difference at *P* < 0.05 compared with the WT, based on Student’s *t*-test.

Of the measured free sphingolipids, the level of total LCBs was 32% higher in β*-OE* than in WT (**Figure 7A**). The level of total LCBPs tended to be lower in β*-KO* than in WT (**Figure 7B**). Of the measured complex sphingolipids, the most abundant classes were GIPC (50%), followed by GlcCer (37%), Cer (8%), and hCer (5%; **Figure 7C**). The level of total Cer tended to be lower while the levels of total GlcCer and total GIPC tended to be higher in β*-OE* than in WT (**Figure 7C**). The total content of complex sphingolipids tended to be slightly higher in β*-OE* than in WT (**Figure 7D**).

## DISCUSSION

These data show that overexpression of *pPLAIII*β results in a 52% increase and knockout mutant has a 26% decrease of 3-KS, the product of the first step of sphingolipid synthesis. Overexpression of *pPLAIII*β leads to increase of several complex sphingolipid species with saturated long chain base and saturated fatty acid chains, such as Cer d18:0–16:0 (90%), Cer t18:0–16:0 (112%), hCer d18:0–h16:0 (72%), GlcCer d18:0–h20:0 (379%), and GIPC t18:0–h16:0 (24%). The total amount of each complex sphingolipid class has no significant difference between WT and *pPLAIII*β-overexpression plants. It is not clear how the overexpression of *pPLAIII*β leads to the alteration of sphingolipid homeostasis.

**FIGURE 8:**
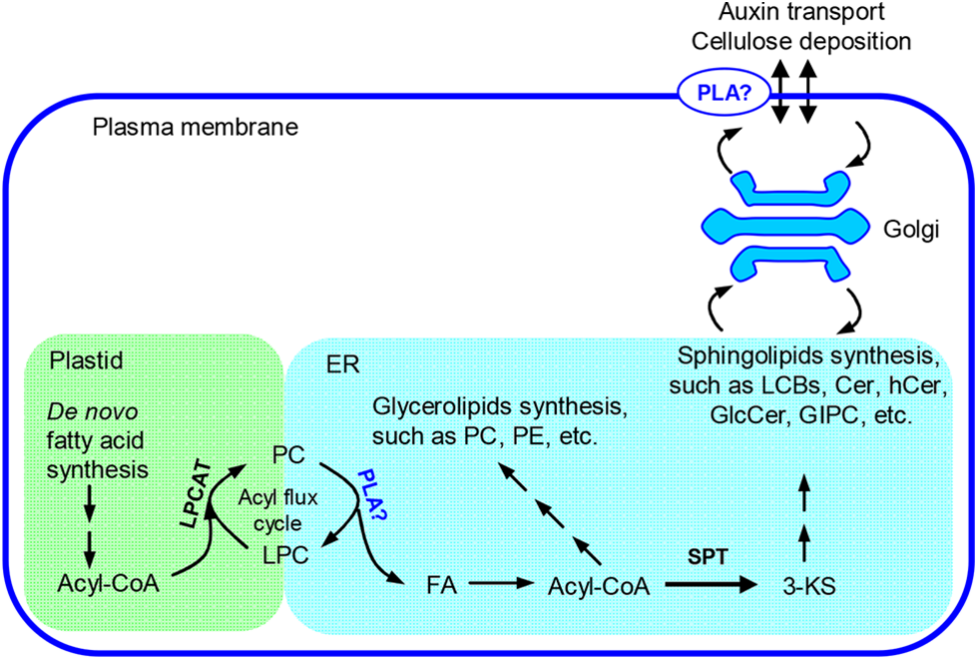
**Proposed role of pPLAIIIβ on sphingolipid synthesis.** In plants, fatty acids are primarily synthesized in plastids and need to be transferred to the ER for assembly of glycerolipids, such as PC and PE, as well as sphingolipids, such as long chain bases(LCBs), Cer, hCer, and GlcCer (Bates et al., 2013; Markham et al., 2013). The synthesis of GIPC takes place at Golgi apparatus (Markham et al., 2013). An acyl flux cycle was proposed for the trafficking of fatty acids from plastids to ER, in which the synthesis of PC was catalyzed by LPCAT and the hydrolysis of PC by PLAs (Lands, 1960; Wang et al., 2012). pPLAIIIβ could be one type of PLA that participates in the acyl flux cycle and contributes to synthesis of the complex membrane lipids. 3-KS, 3-keto-sphinganine; Cer, ceramide; ER, endoplasmic reticulum; FA, free fatty acids; GlcCer, glucosylceramide; GIPC, glycosylinositolphosphoceramide; hCer, hydroxyceramide; LPC, lysophosphatidylcholine; LPCAT, LPC acyltransferase; PC, phosphatidylcholine; PE, phosphatidylethanolamine; PLA, phospholipase A; SPT, serine-palmitoyltransferase.

pPLAIIIβ and pPLAIIIδ can hydrolyze PC and generate LPC and FA (Li et al., 2011, 2013a). It is implicated that *pPLAIIIs* are involved in the fatty acyl trafficking from plastids to ER (Li et al., 2013a). Overexpression of *pPLAIII*β may promote the fatty acyl flux from plastids to ER and enlarge certain fatty acyl pools that provide fatty acyl substrates for sphingolipid synthesis. We observed that the level of 3-KS, the precursor of sphingolipid synthesis, was significantly increased in *pPLAIII*β-overexpression plants. The alteration of this critical first step of sphingolipid synthesis could lead to the observed changes in sphingolipid homeostasis (**Figure 8**).

Sphingolipids are the major components of the plasma membrane (Sperling et al., 2004). Changes in sphingolipid homeostasis may alter structure integrity of raft-like domains in the plasma membrane and therefore influence cell surface activities, such as lipid trafficking and cell wall metabolism (Mongrand et al., 2004; Borner et al., 2005; Melser et al., 2011). Overexpression of *pPLAIII*β results in a decreased level of cellulose content, a loss of anisotropic cell expansion, and a thinner cell wall (Li et al., 2011). Plasma membrane dynamics contribute significantly to the buildup of the cell wall (Li et al., 2013b). It could be possible that the altered sphingolipid homeostasis in *pPLAIII*β mutants impairs cell membrane activities which consequently results in a reduced cellulose production and plant growth.

Multiple lines of evidence suggest that *pPLAIII*β plays a role in auxin transport. In the early seedling stage, some auxin-related phenotypes were shown for *pPLAIII*β mutants, such as slightly longer roots and hypocotyls in *pPLAIII*β*-KO* mutants and much shorter roots and hypocotyls, as well as smaller leaves in *pPLAIII*β*-OE* (Li et al., 2011). Reduced lobe formation in the interdigitating pattern of leaf epidermis cells in *pPLAIII*β*-OE* resembles those observed in auxin receptor mutant *abp1* (auxin-binding protein1; Xu et al., 2011). In addition, the induction of early auxin response genes was delayed in *pPLAIII*β*-KO* mutants (Labusch et al., 2013).

The altered sphingolipid composition in *pPLAIII*β mutants may disturb the auxin transport. Alteration of *pPLAIII*β expression changed the levels of sphingolipid metabolites, particularly species with saturated long chain base and saturated fatty acyl chain, such as Cer d18:0–16:0 and t18:0–16:0, hCer d18:0–h16:0, and GIPC d18:0–h16:0, and t18:0–h16:0 (**Figures 3–6**). Disruption of CS genes diminished the production of sphingolipids with very long chain fatty acids (>18C), impaired the auxin transport, and led to auxin defective phenotypes (Markham et al., 2011). Important functions of sphingolipids on the trafficking of auxin carriers PIN1 (PIN-Formed 1) and AUX1 (Auxin Resistant 1) were evidenced by detailed analyses of an auxin transporter, ATP-binding cassette B19 (ABCB19) auxin transporter (Yang et al., 2013). Sphingolipids are essential components of membrane microdomains or lipid rafts where they attract a unique subset of proteins and together are transported to the plasma membrane (Klemm et al., 2009). The presence of very long chain fatty acids and saturated long carbon chains in sphingolipids can increase their hydrophobicity and the transition from a fluid to a gel phase, which are required for microdomain or lipid raft formation. The altered levels of sphingolipids with saturated acyl chains in *pPLAIII*β mutants may impact the membrane physical properties, the membrane functions on auxin transport, the induction of auxin response gene expression, and subsequently the auxin-related growth.

In summary, our data show that overexpression of *pPLAIII*β alters sphingolipid homeostasis. Our study implies that *pPLAIII*β may influence the substrate availability of the first step of sphingolipid synthesis, which may alter the sphingolipid homeostasis, change the membrane integrity, and eventually impede plant growth.

## MATERIALS AND METHODS

### PLANT GROWTH CONDITION AND GENERATION OF OVEREXPRESSION MUTANTS

Plants were grown in growth chambers with a 12 h light/12 h-dark cycle, at 23/21°C, in 50% humidity, under 200 μmol m^-2^ sec^-1^ of light intensity, and watered with fertilizer once a week. The WT and the mutant *Arabidopsis* are in Columbia-0 background (Col-0). To overexpress *pPLAIII*β, the genomic sequence of *pPLAIII*β was obtained by PCR using Col-0 *Arabidopsis* genomic DNA as a template. The genomic DNA was cloned into the pMDC83 vector before the GFP-His coding sequence. The expression was under the control of the 35S cauliflower mosaic virus promoter. The detailed procedure to generate overexpression lines of *pPLAIII*β was described previously (Li et al., 2011).

### SPHINGOLIPID PROFILING

Leaves from 4 week old plants were harvested and immediately immersed into liquid nitrogen. The frozen samples were lyophilized and stored at -80°C before sphingolipid extraction. Approximately 30 mg of freeze-dried *Arabidopsis* leaves was processed for the sphingolipid profiling using mass spectrometry. The detailed protocols of sphingolipid extraction, detection, and quantification were described previously (Markham and Jaworski, 2007; Markham, 2013).

## Conflict of Interest Statement

The Review Editor Dr. Daniel Hofius declares that, despite having collaborated with author Jonathan E. Markham, the review process was handled objectively. The Review Editor Dr. Günther F. E. Scherer declares that, despite having collaborated with authors Maoyin Li and Xuemin Wang, the review process was handled objectively. The authors declare that the research was conducted in the absence of any commercial or financial relationships that could be construed as a potential conflict of interest.
